# Factores sociodemográficos, alimentarios y condiciones de salud: determinantes de la malnutrición en personas mayores de Colombia

**DOI:** 10.1590/0102-311XES189423

**Published:** 2024-09-23

**Authors:** Alejandro Estrada-Restrepo, Gloria Cecilia Deossa-Restrepo, María Victoria Benjumea-Rincón, Nubia Amparo Giraldo-Giraldo

**Affiliations:** 1 Escuela de Nutrición y Dietética, Universidad de Antioquia, Medellín, Colombia.; 2 Grupo de Investigación en Demografía y Salud, Universidad de Antioquia, Medellín, Colombia.; 3 Grupo de Investigación en Socioantropología de la Alimentación, Universidad de Antioquia, Medellín, Colombia.; 4 Grupo de Investigación en Alimentación y Nutrición Humana, Universidad de Antioquia, Medellín, Colombia.

**Keywords:** Desnutrición, Sobrepeso, Obesidad Abdominal, Antropometría, Adulto Mayor, Malnutrition, Overweight, Abdominal Obesity, Anthropometry, Elderly, Desnutrição, Sobrepeso, Obesidade Abdominal, Antropometria, Pessoa de Idade

## Abstract

El objetivo del estudio fue estimar la prevalencia de malnutrición por indicadores antropométricos agrupados y describir los factores sociodemográficos, alimentarios y condiciones de salud determinantes de malnutrición en personas mayores colombianas. Se realizó un análisis secundario del estudio *Salud, Bienestar y Envejecimiento* (SABE) Colombia, 2015. Incluyó 23.694 personas ≥ 60 años. La malnutrición por exceso se definió agrupando dos indicadores: índice de masa corporal (IMC) y circunferencia de cintura; el déficit de peso se definió agrupando el IMC y las circunferencias de brazo y pantorrilla. Para asociar la malnutrición con variables sociodemográficas, alimentarias y condiciones de salud se usó la prueba chi-cuadrado y para determinar la heterogeneidad de la malnutrición se realizó un análisis de clases latentes. El exceso de peso fue 31,9%; mientras que el déficit de peso según IMC y circunferencia de pantorrilla fue 7,9%, e incrementó a 18,8%, al tener en cuenta además la circunferencia del brazo. Se generaron cinco clases latentes para malnutrición, clase 1: sin exceso de peso y con deterioro en condiciones de salud; clase 2: sin déficit de peso y con deterioro en condiciones de salud; clase 3: sin malnutrición ni deterioro en condiciones de salud; clase 4: exceso de peso y multimorbilidad, y clase 5: bajo consumo de alimentos proteicos sin déficit ni exceso de peso. Se concluye que existe una prevalencia de malnutrición elevada en las personas mayores, representando más el exceso que el déficit. Tanto los factores sociodemográficos, alimentarios y condiciones de salud, se asocian de forma diferente al exceso que al déficit de peso.

## Abstract (en)

This study aimed to estimate the prevalence of malnutrition using clustered anthropometric indicators and to describe the sociodemographic and dietary factors and health conditions that determine malnutrition in elderly Colombians. This was a secondary analysis of the study *Health, Well-being and Ageing* (SABE) Colombia, 2015. The survey included 23,694 people aged ≥ 60 years. Malnutrition excess was defined by clustering two indicators: body mass index (BMI) and waist circumference; weight deficit was defined by clustering BMI and arm and calf circumferences. The chi-square test was used to associate malnutrition with sociodemographic variables, dietary and health conditions, and to determine the heterogeneity of malnutrition, a latent class analysis was performed. Overweight was 31.9%, whereas underweight, according to BMI and calf circumference, was 7.9%, and increased to 18.8% when arm circumference was also taken into account. Five latent classes of malnutrition were generated - class 1: no overweight and deteriorated health conditions; class 2: no weight deficit and deteriorated health conditions; class 3: no malnutrition and deteriorated health conditions; class 4: overweight and multimorbidity; and class 5: low protein food intake without being underweight or overweight. It is concluded that a high prevalence of malnutrition in older adults exists, with excess rather than deficit. Sociodemographic and dietary factors and health conditions are associated differently with overweight and underweight.

## Resumo

O objetivo do estudo foi estimar a prevalência da desnutrição por meio de indicadores antropométricos agrupados e descrever os fatores sociodemográficos, alimentares e condições de saúde determinantes da desnutrição em idosos colombianos. Foi realizada uma análise secundária do estudo *Saúde, Bem-estar e Envelhecimento* (SABE) Colômbia, 2015. A pesquisa incluiu 23.694 pessoas com idade ≥ 60 anos. O excesso de desnutrição foi definido pelo agrupamento de dois indicadores: índice de massa corporal (IMC) e circunferência da cintura; o déficit de peso foi definido pelo agrupamento do IMC e das circunferências do braço e da panturrilha. Para associar a desnutrição a variáveis sociodemográficas, condições alimentares e de saúde, foi usado o teste do qui-quadrado e, para determinar a heterogeneidade da desnutrição, foi realizada uma análise de classe latente. O excesso de peso foi de 31,9%, enquanto o baixo peso, de acordo com o IMC e a circunferência da panturrilha, foi de 7,9%, e aumentou para 18,8% quando a circunferência do braço também foi levada em conta. Foram geradas cinco classes latentes para a desnutrição - classe 1: sem excesso de peso e condições de saúde deterioradas; classe 2: sem déficit de peso e condições de saúde deterioradas; classe 3: sem desnutrição e condições de saúde deterioradas; classe 4: excesso de peso e multimorbidade; e classe 5: baixa ingestão de alimentos proteicos sem déficit de peso ou excesso de peso. Conclui-se que há uma alta prevalência de desnutrição em idosos, com excesso em vez de déficit. Fatores sociodemográficos, alimentares e condições de saúde estão associados de forma diferente ao sobrepeso e ao baixo peso.

## Introducción

La malnutrición por déficit es un estado secundario a un consumo insuficiente de calorías/nutrientes que conlleva a alteración de la composición corporal, disminución de la masa libre de grasa y de la masa celular corporal, lo que afecta la función física, mental y también el resultado clínico de las enfermedades ^1^ ^,^ ^2^; en contraste, la malnutrición por exceso se presenta por un consumo excesivo de calorías/nutrientes o un desbalance en la proporción de los mismos, considerado como un problema frecuente en las personas mayores ^3^ ^,^ ^4^. Las cifras mundiales de desnutrición de las personas mayores varían desde 3% en la comunidad, hasta 29% en los institucionalizados. En Estados Unidos la prevalencia de obesidad en las personas mayores supera el 30% en ambos sexos ^5^. En Antioquia (Colombia), según el *Perfil Alimentario y Nutricional* del 2019, 38,5% de las personas mayores presentan exceso de peso y 20,3% déficit ^6^.

La antropometría es un método válido para evaluar el estado nutricional de las personas mayores, en cualquier ámbito. El índice de masa corporal (IMC) es de uso común y se relaciona con enfermedades crónicas no transmisibles ^7^, fragilidad y disfuncionalidad cuando se clasifica en exceso ^8^. En contraste, el déficit de peso se asocia con mayor frecuencia de mortalidad y más días de estancia hospitalaria ^2^ ^,^ ^9^. Por su parte, la circunferencia de la cintura (CC) es una medida complementaria del IMC, estima la adiposidad central y se asocia con morbilidad y mortalidad por todas las causas ^10^, con enfermedades crónicas no transmisibles ^7^, y con síndromes geriátricos ^8^.

En las personas mayores, la grasa corporal incrementa y su distribución cambia con tendencia a una acumulación mayor en la región central y menor en las extremidades ^7^; por el contrario, la masa muscular y la fuerza decrecen y se relacionan con la dependencia durante el envejecimiento ^11^. La calidad e intensidad de estos cambios están determinados por factores genéticos, ambientales y de salud ^12^ ^,^ ^13^. Las circunferencias del brazo (CB) y de la pantorrilla (CP) reflejan el estado de la grasa subcutánea y de la masa muscular, siendo la CP un buen indicador de masa muscular apendicular ^14^ ^,^ ^15^ ^,^ ^16^. Mientras que una baja CB, se relaciona con un mayor riesgo de morbilidad y muerte en esta población ^15^. Si bien cada una de estas medidas antropométricas ofrece ventajas para la determinación del estado nutricional de la población de personas mayores, el uso combinado mejora el diagnóstico.

Respecto a los factores asociados con el estado nutricional, en una revisión sistemática ^17^ en la cual se agruparon los estudios según el tipo de envejecimiento de los participantes en “exitoso”, “usual” y “acelerado”, encontraron que los aspectos sociales, la ingesta de alimentos y las enfermedades relacionadas, fueron más severos en la categoría de envejecimiento acelerado. Aunque los estudios referentes al estado civil y el nivel educativo no son consistentes, en esta revisión sistemática el no estar casado se asoció con riesgo o malnutrición en la categoría de envejecimiento exitoso, un comportamiento similar se evidenció con un nivel educativo bajo.

En un metaanálisis ^18^, se reportó mayor probabilidad de malnutrición en las mujeres comparadas con los hombres, posiblemente porque ellas tienen mayor expectativa de vida y unas condiciones socioeconómicas más desfavorables a medida que envejecen. En congruencia con lo anterior, un estudio observacional con personas mayores de la ciudad de Medellín (Colombia) ^19^, halló mayor probabilidad de riesgo de desnutrición y desnutrición en mujeres, en personas ≥ 75 años, de estrato bajo, sin estudios o con primaria y en personas mayores de la zona rural.

Por lo anterior, se plantearon como objetivos: (1) estimar la prevalencia de malnutrición por indicadores antropométricos agrupados para déficit y exceso de peso y, (2) analizar los factores sociodemográficos, alimentarios y condiciones de salud determinantes de la malnutrición en las personas mayores de Colombia.

### Material y métodos

Estudio transversal que incluyó adultos ≥ 60 años, no institucionalizados y que vivían en zonas urbanas y rurales, evaluados en el estudio *Salud, Bienestar y Envejecimiento* (SABE) Colombia, 2015.

En dicha encuesta se aplicó muestreo por conglomerados, multietápico, probabilístico y estratificado. Con representatividad nacional, para las cinco regiones, Atlántica, Oriental, Orinoquía y Amazonía, Central y Pacífica, y para cuatro grandes ciudades del país: Bogotá, Medellín, Cali y Barranquilla. La información más detallada sobre el estudio fue descrita por Goméz et al. ^20^. Para esta investigación se excluyeron aquellas personas mayores con valores perdidos, extraños o biológicamente imposibles en las medidas antropométricas.

### Variables

#### Dependientes

La malnutrición (exceso y déficit de peso) se definió a partir de diferentes criterios. El exceso de peso se clasificó cuando se presentó a la vez un IMC ≥ 28,0kg/m^2^ y una CC > 102cm para hombres y > 88cm para mujeres (obesidad abdominal). El déficit de peso se estableció cuando la personas mayores presentaba simultáneamente un IMC ≤ 23kg/m^2^, una CP < 31cm y una CB (cm) en déficit según la clasificación de la *III Encuesta Nacional de Salud y Nutrición de los Estados Unidos* (NHANES III, por sus siglas en inglés) que considera normalidad cifras entre el percentil 25 y 75, en el presente estudio una CB (cm) en déficit se clasificó por edad y sexo, así: en el grupo de 60-69 años para hombres, valores inferiores a 30,6cm y para mujeres a 28,3cm; en el grupo de 70-79 años, para hombres cifras menores a 29,3cm y para mujeres a 27,4cm; y en el grupo de 80 y más, para hombres valores inferiores a 27,3cm y 25,5cm para mujeres ^21^.

El IMC (kg/m^2^) se clasificó de acuerdo con los criterios de la Organización Panamericana de la Salud (OPS) así: ≤ 23,0kg/m^2^ (Delgadez); > 23,0kg/m^2^ a < 28,0kg/m^2^ (normalidad); ≥ 28,0kg/m^2^ a < 32,0kg/m^2^ (sobrepeso); ≥ 32,0kg/m^2^ (obesidad) ^22^. La CC se clasificó con los puntos de corte del Instituto Nacional de Salud de Estados Unidos (NHI, por sus siglas en inglés), valores > 102cm para hombres y > 88cm para mujeres se consideraron como obesidad abdominal ^23^. Para la CP, valores < 31cm se definieron como baja masa muscular y ≥ 31cm como normales ^24^. Por su parte, la CB se clasificó de acuerdo con los valores de referencia de la NHANES III según sexo y edad ^21^.

#### Independientes

(a) Socioeconómicas y demográficas: región (Atlántica, Oriental, Bogotá, Orinoquía y Amazonía, Central y Pacífica), edad (60-69, 70-79, 80 y más); sexo (hombre, mujer); nivel educativo (ninguno, primaria, secundaria, técnico/tecnólogo y universidad/posgrado); estrato socioeconómico (1-6, de acuerdo a las características y condiciones de vida donde se localiza el hogar, donde 1 es el estrato más bajo y 6 el más alto), número de personas con quien vive (0, 1-2, 3-4, 5-6, 7 o más).

(b) Consumo de alimentos: número de comidas completas que toma al día (1, 2, 3 o más); consumo de leche o productos lácteos al menos una vez al día (sí, no); consumo de huevos, frijoles o leguminosas una o dos veces por semana (sí, no); consumo de carne, pescado o aves diariamente (sí, no) y necesidad de ayuda para alimentarse (necesita ayuda, se alimenta solo con dificultad, se alimenta solo sin dificultad).

(c) Morbilidad y estado de salud: número de enfermedades crónicas que tenía al momento de la encuesta y que habían sido previamente diagnosticadas por un médico (0, 1, 2, 3, 4-7).

(d) Hospitalización: hospitalización en el último año, autorreportado (sí, no).

(e) Uso de medicamentos: medicamentos prescritos por médico, consumidos diariamente al momento de la encuesta (0, 1, 2, 3, 4, 5-9, ≥ 10).

El estado funcional en relación con las actividades básicas de la vida diaria se determinó con la escala de Barthel ^25^, la cual mide el desempeño en los ítems de: alimentación, aseo, baño, vestido, deposición, micción, uso del inodoro, traslado silla/cama, deambulación y subir escaleras; esta escala puntúa entre 0 y 100, clasificando como dependiente a quien presentaba puntajes < 100. La presencia de síntomas depresivos se determinó con la escala de valoración geriátrica de Yesavage et al. ^26^, la cual va de 0 a 15 y se utilizó un punto de corte de 6 o más para considerar la presencia de síntomas depresivos; se incluyó también la escala del índice de valoración de salud oral geriátrica (GOHAI, por sus siglas en inglés), indicador subjetivo de salud bucal, que evalúa la percepción de problemas funcionales y los impactos psicosociales asociados con la condición de salud bucal, tiene un puntaje total que oscila entre 12 y 60, clasificando como baja calidad de vida relacionada con la salud bucal, puntajes < 51, moderada entre 51 y 56 y alta ≥ 57 puntos ^27^.

Las medidas antropométricas fueron tomadas por personal entrenado y capacitado especialmente para el estudio SABE 2015 ^20^ ^,^ ^28^.

### Análisis estadístico

El análisis estadístico fue desarrollado en SPSS, versión 24 (https://www.ibm.com/), se incluyeron las ponderaciones muestrales en los cálculos y los intervalos de 95% de confianza (IC95%) para tener en cuenta el diseño complejo de la encuesta. Las variables categóricas fueron presentadas como frecuencias relativas y las numéricas como promedios y desviación estándar. La asociación de cada uno de los criterios de malnutrición con las demás variables se realizó mediante la prueba chi-cuadrado de independencia con nivel de significancia estadística de 5%. Se efectuó análisis de clases latentes (ACL) ^29^ ^,^ ^30^ que permitió determinar número, tamaño y características de grupos “latentes” para identificar la heterogeneidad de la malnutrición en la personas mayores; para ello, se incluyeron edad, sexo, sintomatología depresiva, GOHAI, dependencia, número y frecuencia de comidas y productos consumidos, número de enfermedades, exceso y déficit de peso. Este análisis se efectuó en R, versión 4.1.3 (http://www.r-project.org), mediante el paquete poLCA 3.5.2 ^31^. Para determinar el número óptimo de clases latentes y el modelo que mejor ajustó, se usó el estadístico de verosimilitud G2, el criterio de información bayesiano (BIC, por sus siglas em inglés) y la interpretabilidad de los parámetros de solución del modelo, con especial atención en el significado interpretativo de los perfiles de respuesta para cada una de las clases, a las que se les dio una denominación específica según las probabilidades resultantes en cada variable estudiada.

### Consideraciones éticas

Este estudio se desarrolló de acuerdo con los principios de la Declaración de Helsinki y la Resolución 008430 de 1993 del Ministerio de Salud de Colombia. Los comités de bioética de la Universidad de Caldas (Acta nº CBCS-021-14) y Universidad del Valle (Actas nº 09-014 y O11-015) aprobaron todos los procedimientos del estudio SABE 2015.

## Resultados

Los resultados de los 23.694 personas mayores estudiadas según variables sociodemográficas y alimentarias, se presentan en la Tabla 1. El promedio de la edad fue 69,8 ± 7,9, la mayoría de las personas evaluadas fueron mujeres (54,5%), de edades de 60-69 años (56,9%), el 68,1% pertenecían a estratos 1 y 2, 70% tenían nivel educativo máximo en primaria; el 70,1% reportó un consumo de tres o más comidas completas al día, y más del 98% manifestó alimentarse solo sin dificultad.

**Tabla 1:** Características sociodemográficas e ingesta de alimentos. Estudio *Salud, Bienestar y Envejecimiento* (SABE) Colombia, 2015.

Características	n	%
Sexo		
Hombre	10.112	45,5
Mujer	13.582	54,5
Edad (años) *		
60-69	12.101	56,9
70-79	7.720	30,2
80 y más	3.873	12,9
Nivel educativo		
Ninguno	5.229	16,6
Primaria	13.462	53,4
Secundaria	3.448	19,2
Técnico/Tecnólogo	667	4,5
Universidad/Posgrado	795	6,3
Número de personas con que viven		
0	2.220	9,2
1-2	10.161	42,7
3-4	6.421	29,2
5-6	3.242	12,9
7 o más	1.650	6,0
Estrato socioeconómico		
1	10.313	28,4
2	9.033	39,7
3	3.611	25,3
4	570	4,6
5 y 6	167	2,0
Número de comidas completas que toma al día		
1	433	2,2
2	5.107	27,7
3 o más	13.444	70,1
Consumo de leche o lácteos al menos una vez al día		
No	5.370	24,4
Sí	13.613	75,6
Consumo de huevos, frijoles o leguminosas una o dos veces por semana		
No	1.758	8,1
Sí	17.237	91,9
Consumo de carne, pescado o aves diariamente		
No	4.125	20,0
Sí	14.850	80,0
Necesidad de ayuda para alimentarse		
Necesita ayuda	125	0,5
Se alimenta solo con dificultad	173	0,9
Se alimenta solo sin dificultad	23.396	98,6

Nota: el porcentaje se ponderó a partir de los pesos muestrales del diseño de muestreo complejo de la encuesta.

* Média ± desviación padrón: 69,8 ± 7,9.

**Figura 1:**
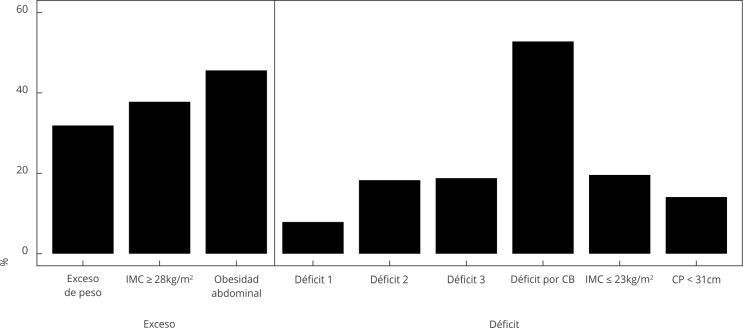
Prevalencia de malnutrición en adultos mayores de Colombia, según indicadores antropométricos individuales y agrupados.

CB: circunferencia del brazo; CP: circunferencia de pantorrilla; IMC: índice de masa corporal. Nota: Déficit 1: IMC ≤ 23kg/m^2^ y CP < 31cm; Déficit 2: IMC ≤ 23kg/m^2^ y déficit por CB; Déficit 3: CP < 31cm o IMC ≤ 23kg/m^2^ y déficit por CB; Obesidad abdominal: circunferencia de cintura (CC) > 88cm para mujeres y > 102cm para hombres; Exceso de peso: IMC ≥ 28kg/m^2^ y obesidad abdominal.

En la Figura 1 y en la Tabla 2 se observa el comportamiento de la malnutrición en las personas mayores; en ellos se destacaron el exceso de peso por la CC y el déficit de peso por la CB. En 31,9% se encontró exceso de peso (IMC y CC); y en 18,8% se presentó déficit de peso (IMC + CP + CB) (Figura 1). El exceso de peso fue mayor en mujeres; en estratos altos y con mayor escolaridad; entre los más jóvenes; con alto índice de GOHAI; dependientes; que consumían solo una comida al día; presentaban mayor número de enfermedades y consumían más medicamentos (p < 0,05); mientras que el déficit de peso fue mayor en hombres; de estrato uno; de más edad; sin ningún nivel educativo; conviviendo con 7 o más integrantes; con bajo índice de GOHAI; sin consumo de leche o productos lácteos, carne, pescado y aves diariamente; además, en quienes requerían ayuda para alimentarse y no presentaban o reportaron solo una enfermedad (p < 0,05) (Tabla 2).

**Tabla 2:** Malnutrición por indicadores agrupados y características de interés. Estudio *Salud, Bienestar y Envejecimiento* (SABE) Colombia, 2015.

Características	Exceso de peso corporal *					Déficit de peso corporal **				
	Total	Si		IC95%	Valor de p ***	Total	Si		IC95%	Valor de p ***
	n	n	%			n	n	%		
Total	19.841	5.964	31,9	30,1-33,7		20.460	4.491	18,8	17,6-20,0	
Región					< 0,001					< 0,001
Atlántico	5.062	1.424	31,6	27,2-36,3		5.198	1.472	24,4	21,8-27,3	
Oriental	3.093	1.002	32,3	28,2-36,7		3.207	610	15,5	12,8-18,7	
Orinoquia y Amazonia	1.242	397	38,9	28,6-50,3		1.262	210	13,7	9,1-20,2	
Bogotá	1.712	635	35,0	29,6-40,8		1.761	229	13,9	10,8-17,6	
Central	5.205	1.503	30,1	27,2-33,1		5.391	1.161	19,5	17,2-22,0	
Pacífica	3.527	1.003	30,7	27,7-33,8		3.641	809	20,4	17,9-23,1	
Estrato socioeconómico					< 0,001					< 0,001
1	8.675	2.268	27,0	24,9-29,2		8.931	2.431	25,3	23,3-27,4	
2	7.604	2.523	34,1	31,4-36,9		7.843	1.487	17,2	15,4-19,1	
3	2.980	997	32,5	29,2-36,0		3.080	468	14,2	11,9-17,0	
4	457	137	31,3	19,8-45,7		477	83	16,9	10,7-25,7	
5 y 6	125	39	47,3	22,9-73,0		129	22	19,6	8,9-37,7	
Sexo					< 0,001					< 0,001
Hombre	8.850	1.483	18,5	16,5-20,7		9.029	2.471	23,2	21,3-25,2	
Mujer	10.991	4.481	43,8	41,2-46,4		11.431	2.020	14,8	13,4-16,4	
Edad (años)					< 0,001					< 0,001
60-69	10.815	3.615	33,2	30,7-35,8		11.097	2.082	17,4	15,8-19,2	
70-79	6.462	1.831	31,4	28,7-34,2		6.668	1.552	19,0	17,1-21,0	
80 y +	2.564	518	24,9	21,4-28,7		2.695	857	25,9	22,7-29,4	
Nivel educativo					< 0,001					< 0,001
Ninguno	4.121	1.012	28,4	25,1-31,9		4.266	1.268	24,2	21,5-27,2	
Primaria	11.381	3.530	32,9	30,7-35,2		11.716	2.486	17,9	16,5-19,4	
Secundaria	2.981	977	30,2	27,0-33,7		3.081	537	19,2	16,1-22,7	
Técnico/Tecnólogo	599	224	30,7	23,2-39,3		613	84	13,2	8,2-20,6	
Universidad/Posgrado	699	203	36,5	24,4-50,5		721	101	15,6	10,0-23,6	
Número de personas con quien viven					< 0,001					< 0,001
0	1.877	482	28,6	24,4-33,1		1.941	475	20,5	17,5-24,0	
1-2	8.559	2.654	33,6	31,0-36,2		8.807	1.804	16,9	15,2-18,7	
3-4	5.359	1.648	31,0	27,2-35,1		5.531	1.176	19,6	17,1-22,3	
5-6	2.676	800	32,4	28,1-37,1		2.767	637	18,2	15,4-21,4	
7 o más	1.370	380	27,6	22,7-33,1		1.414	399	26,9	21,8-32,7	
Presencia de síntomas depresivos					0,042					0,552
No	7.095	2.293	33,6	30,4-37,0		7.312	1.420	15,8	14,0-17,7	
Sí	9.518	2.935	32,0	29,7-34,3		9.775	1.934	17,7	16,0-19,5	
Índice GOHAI					< 0,001					< 0,001
Alto	5.424	1.804	36,4	32,5-40,4		5.543	895	13,9	11,9-16,2	
Moderado	5.537	1.792	31,6	28,6-34,8		5.707	1.086	16,1	14,2-18,2	
Bajo	5.644	1.631	30,0	27,3-32,9		5.829	1.372	20,6	18,2-23,2	
Dependencia					< 0,001					0,017
Independencia	16.420	4.783	30,6	28,6-32,7		16.878	3.651	18,7	17,4-20,1	
Dependencia	3.421	1.181	38,1	34,5-41,9		3.582	840	19,1	16,6-21,8	
Número de comidas completas que toma al día					< 0,001					
1	378	154	49,8	39,4-60,2		389	77	15,8	11,0-22,2	
2	4.481	1.614	35,7	32,5-38,9		4.599	804	14,8	12,6-17,3	
3 o más	11.779	3.466	31,2	28,7-33,8		12.125	2.479	17,5	16,0-19,1	
Consumo de leche o lácteos al menos una vez al día					< 0,001					< 0,001
No	4.707	1.281	29,8	26,3-33,5		4.819	1.088	20,4	18,1-22,9	
Sí	11.932	3.953	33,7	31,3-36,1		12.296	2.276	15,6	14,1-17,2	
Consumo de huevos, frijoles o leguminosas una o dos veces por semana					0,729					0,125
No	1.529	475	32,8	28,1-37,9		1.574	332	19,0	15,3-23,3	
Sí	15.122	4.763	32,8	30,7-34,9		15.552	3.030	16,6	15,3-18,0	
Consumo de carne, pescado o aves diariamente					< 0,001					< 0,001
No	3.627	946	27,4	24,1-31,0		3.732	883	21,9	19,0-25,0	
Sí	13.005	4.286	34,1	31,9-36,5		13.375	2.475	15,3	14,0-16,8	
Necesidad de ayuda para alimentarse					0,713					0,011
Necesita ayuda	21	6	18,4	7,3-39,3		21	6	37,1	17,1-62,6	
Se alimenta solo con dificultad	63	16	32,8	14,8-57,8		65	24	26,0	13,2-44,8	
Se alimenta solo sin dificultad	19.757	5.942	31,9	30,1-33,7		20.374	4.461	18,7	17,5-20,0	
Número de enfermedades					< 0,001					< 0,001
0	5.229	686	12,9	10,2-16,0		5.370	1.787	28,4	25,7-31,3	
1	6.089	1.554	27,0	23,6-30,7		6.261	1.476	22,4	19,9-25,2	
2	4.519	1.658	34,4	31,2-37,7		4.664	762	14,1	12,0-16,4	
3	2.523	1.185	50,7	46,2-55,1		2.605	331	8,5	7,0-10,3	
4-7	1.480	880	67,6	62,3-72,4		1.559	135	6,7	4,8-9,1	
Número de medicamentos prescriptos por médico					< 0,001					< 0,001
0	6.338	1.238	19,6	17,4-22,1		6.515	1.947	27,0	24,5-29,7	
1	2.750	749	27,9	23,3-33,0		2.826	665	23,5	20,2-27,2	
2	2.751	832	31,0	26,1-36,4		2.860	598	17,9	14,8-21,6	
3	2.652	935	32,7	28,8-36,9		2.743	508	16,1	12,9-19,9	
4	1.991	756	45,1	37,8-52,6		2.055	336	10,5	8,4-13,2	
5-9	3.138	1.354	43,9	40,1-47,7		3.223	409	10,9	8,9-13,4	
≥ 10	221	100	53,9	40,4-66,8		238	28	8,7	4,2-17,1	
Hospitalización en el último año					0,052					0,384
No	17.495	5.218	31,5	29,6-33,5		18.024	3.940	18,8	17,5-20,2	
Sí	2.344	745	34,5	30,3-39,0		2.434	551	18,4	15,4-21,8	

IC95%: intervalo de 95% de confianza.

Nota: el porcentaje se ponderó a partir de los pesos muestrales del diseño de muestreo complejo de la encuesta.

* Presencia simultánea de exceso por índice de masa corporal (IMC) y obesidad abdominal por circunferencia de cintura (CC);

** Presencia de IMC ≤ 23kg/m^2^ y circunferencia de pantorrilla (CP) < 31cm o circunferencia de brazo (CB) en déficit según *III Encuesta Nacional de Salud y Nutrición de los Estados Unidos* (NHANES III);

*** Prueba de chi-cuadrado de independencia.

**Figura 2:**
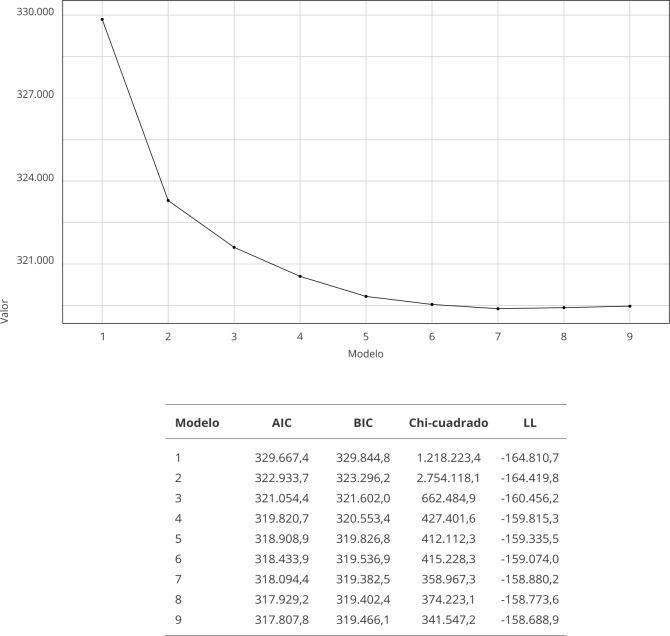
Comparación de modelos latentes.

AIC: criterio de información Akaike; BIC: criterio de información bayesiano; LL: *log-likelihood*.

Finalmente, en el análisis ACL, a partir del BIC, se pudo evidenciar que el punto óptimo de clases fue 5 (Figura 2); se observó además que al adicionar una sexta clase no mejoró el desempeño del modelo y, en cambio, se complejizaba la interpretación de este, corroborado con el aplanamiento de la pendiente a partir de la quinta clase.

### Clases latentes

El análisis de las clases latentes se presenta en la Tabla 3.

**Tabla 3:** Clases latentes y probabilidades condicionales. Estudio *Salud, Bienestar y Envejecimiento* (SABE) Colombia, 2015.

Variables	Clase 1 (15,4%)	Clase 2 (29,1%)	Clase 3 (24,4%)	Clase 4 (18,6%)	Clase 5 (12,5%)
Sexo					
Mujer	0,5141	0,6420	0,2877	0,8562	0,4122
Hombre	0,4859	0,3580	0,7123	0,1438	0,5878
Edad (años)					
60-69	0,2718	0,7324	0,6911	0,5510	0,5765
70-79	0,4725	0,2471	0,2643	0,3396	0,3318
80 y más	0,2557	0,0205	0,0445	0,1094	0,0918
Estrato socioeconómico					
1	0,3286	0,3175	0,5248	0,3528	0,6322
2	0,4038	0,4400	0,3345	0,4270	0,3294
3	0,2158	0,1919	0,1212	0,1940	0,0384
4	0,0422	0,0381	0,0152	0,0212	0,0000
5 y 6	0,0096	0,0126	0,0043	0,0051	0,0000
Presencia de síntomas depresivos					
Síntomas depresivos	0,5148	0,5760	0,6270	0,5385	0,5896
Normal	0,4852	0,4240	0,3730	0,4615	0,4104
Valoración de salud oral geriátrica					
Alto	0,2670	0,4442	0,3530	0,2496	0,1915
Moderado	0,3396	0,3531	0,3338	0,3332	0,2826
Bajo	0,3934	0,2027	0,3132	0,4171	0,5259
Actividades básicas de la vida diaria					
Dependencia	0,2880	0,0091	0,0191	0,3404	0,1560
Independencia	0,7120	0,9909	0,9809	0,6596	0,8440
Número de comidas completas que toma al día					
1	0,0106	0,0150	0,0083	0,0421	0,0548
2	0,1841	0,2376	0,2070	0,3583	0,4340
3 o más	0,8052	0,7474	0,7847	0,5996	0,5112
Consumo de leche o lácteos al menos una vez al día					
Sí	0,8348	0,8472	0,7599	0,7025	0,2148
No	0,1652	0,1528	0,2401	0,2975	0,7852
Consumo de huevos, frijoles o leguminosas una o dos veces por semana					
Sí	0,9315	0,9661	0,9535	0,8548	0,7383
No	0,0685	0,0339	0,0465	0,1452	0,2617
Consumo de carne, pescado o aves diariamente					
Sí	0,8489	0,9069	0,8400	0,7703	0,3227
No	0,1511	0,0931	0,1600	0,2297	0,6773
Número de enfermedades					
0	0,1151	0,1948	0,6041	0,0312	0,3170
1	0,3410	0,3734	0,3223	0,1506	0,3242
2	0,2990	0,2871	0,0710	0,2718	0,2220
3	0,1744	0,1232	0,0025	0,2750	0,0958
4-7	0,0705	0,0215	0,0000	0,2713	0,0409
Exceso de peso					
No	1,0000	0,4696	1,0000	0,1878	0,9223
Sí	0,0000	0,5304	0,0000	0,8122	0,0777
Déficit de peso					
Sí	0,3630	0,0000	0,3991	0,0000	0,2957
No	0,6370	1,0000	0,6009	1,0000	0,7043

Nota: clase 1: sin exceso de peso y con deterioro en condiciones de salud; clase 2: sin déficit de peso y con deterioro en condiciones de salud; clase 3: sin malnutrición ni deterioro en condiciones de salud; clase 4: exceso de peso y multimorbilidad; clase 5: bajo consumo de alimentos proteicos sin déficit ni exceso de peso.

La clase 1 “Sin exceso de peso y con deterioro en condiciones de salud” agrupó 15,4% de las personas mayores y comprendió características como: mujeres, entre 70-79 años, de estrato 2, independientes, con tres o más comidas completas al día, consumían leche o productos lácteos al menos una vez al día, huevos o leguminosas una o dos veces por semana y carne, pescado o aves diariamente, con síntomas depresivos, bajo índice de GOHAI, con presencia de una enfermedad, sin exceso ni déficit de peso.

La clase 2 “Sin déficit de peso y con deterioro en condiciones de salud” agrupó 29,1% de las personas mayores y comprendió características como: mujeres, entre 60-69 años, estrato 2, con síntomas depresivos, alto índice de GOHAI, independientes, con presencia de una enfermedad, que realizaban tres o más comidas completas al día, consumían leche o productos lácteos al menos una vez al día, huevos o leguminosas una o dos veces por semana, carne, pescado o aves diariamente y con exceso de peso y sin déficit de peso.

La clase 3 “Sin malnutrición ni deterioro en condiciones de salud” agrupó el 24,4% de las personas mayores con características como: hombres, entre 60-69 años, de estrato 1, con síntomas depresivos, alto índice de GOHAI, independientes, con tres o más comidas completas al día, consumían leche o productos lácteos al menos una vez al día, huevos o leguminosas una o dos veces por semana, carne, pescado o aves diariamente, sin enfermedades, ni exceso o déficit de peso.

La clase 4 “Exceso de peso y multimorbilidad” agrupó 18,6% de las personas mayores, con características como: mujeres, entre 60-69 años, estrato 2, con síntomas depresivos, bajo índice de GOHAI, independientes, con tres o más comidas completas al día, consumían leche o productos lácteos al menos una vez al día, huevos o leguminosas una o dos veces por semana, carne, pescado o aves diariamente, con dos o más enfermedades, y con exceso de peso.

La clase 5 “Bajo consumo de alimentos proteicos sin déficit ni exceso de peso” agrupó 12,5% de las personas mayores y comprendió características como: hombres, entre 60-69 años, de estrato 1, con síntomas depresivos, bajo índice de GOHAI, independientes, que realizaban tres o más comidas completas al día, no consumían leche o productos lácteos al menos una vez al día, consumían huevos o leguminosas una o dos veces por semana, no consumían carne, pescado o aves diariamente, y a lo sumo tenían una enfermedad, sin exceso o déficit de peso.

## Discusión

Nuestros hallazgos reflejan cifras de malnutrición elevadas en las personas mayores colombianas, con especial énfasis en el exceso de peso, condición más común en mujeres de menor edad, con buena calidad de salud bucal, de estratos más altos, dependientes y con mayor número de enfermedades; mientras que el déficit de peso fue más común en personas mayores de sexo masculino, de edades más avanzadas, de estrato socioeconómico uno, sin ningún nivel educativo, con menor ingesta de alimentos fuentes de proteína, que requieren ayuda para alimentarse y sin presencia de enfermedades. En las cinco clases latentes resultantes, priman las mujeres de menor edad, independientes, de estratos socioeconómicos más bajos y con deterioro en sus condiciones de salud. De las cinco clases obtenidas, tres incluyen déficit o exceso de peso como variable integrante de la malnutrición de las personas mayores.

Al comparar nuestros resultados con los de otros investigadores, se encuentran cifras mayores de exceso de peso en personas mayores chilenas 52,7% (colombianos 31,9%) y menores de bajo peso (chilenos: 9,9% *vs*. colombianos: 18,8%) ^32^ utilizando los mismos puntos de corte que nuestro estudio. Es de anotar que, al considerar el exceso de peso en las personas mayores, únicamente con el IMC, las cifras fueron 5,8 puntos porcentuales superiores a la encontrada cuando se combina con la CC, con lo que mejora la captación de personas mayores con riesgo de síndrome metabólico, entre otras enfermedades crónicas ^7^.

El exceso de peso corporal fue la condición de malnutrición más prevalente en las personas mayores, llegando casi a duplicar el déficit de peso. Este comportamiento también se halló en estudios con personas mayores en México ^33^, Perú ^34^ y Brasil ^35^. La frecuencia de exceso de peso elevada puede tener efectos deletéreos en este grupo de edad, generar dificultades en la movilidad y exacerbar signos y síntomas de enfermedades que son más comunes en etapas avanzadas de la vida ^36^. Un estudio en Sudáfrica encontró que las mujeres mayores de 50 años presentaron un riesgo más elevado de desarrollar obesidad central (33%) en comparación con los hombres de la misma edad (20%), según CC y relación cintura/cadera ^37^, hallazgos similares a los nuestros; sin embargo, encontraron que los hombres de mayor edad, con mejores condiciones socioeconómicas, presentaron más riesgo de obesidad ^37^; contrario a lo que se observó en las personas mayores colombianas, donde las mujeres y personas de menor edad, presentaron mayor frecuencia de exceso de peso. Estas diferencias podrían tener una explicación posiblemente desde los condicionantes económicos y socioculturales y la presencia de depresión. Por otro lado, los resultados de un estudio en São Paulo, Brasil ^38^, mostraron mayor proporción de obesidad por cintura y por relación cintura/cadera en las mujeres y en personas con polifarmacia, así como mayor frecuencia de obesidad por cintura en aquellos de menor edad (72-79 años), hallazgos similares al presente estudio, a pesar que el de Brasil fue en una población de más edad que la nuestra, entre 72 y 102 años con promedio de edad de 80,8 años y mayoritariamente femenina (70%).

El déficit de peso, determinado simultáneamente por varios indicadores antropométricos, fue menos prevalente en las personas mayores colombianas, que el exceso de peso corporal, incluso cuando este último se estableció por IMC solo o en combinación con la CC. Referente al déficil de peso, los procesos de enfermedad, así como factores socioeconómicos, están marcadamente relacionados con el; nuestro estudio encontró mayor porcentaje de déficit de peso en personas mayores de estrato 1, ningún nivel educativo y en hogares con mayor número de personas. Estas condiciones pueden llevar a una disminución en la cantidad y calidad de alimentos consumidos y exacerbar la inseguridad alimentaria y nutricional en los hogares. Al respecto, la *Encuesta Nacional de Situación Nutricional en Colombia* en el año 2015, reportó una prevalencia de inseguridad alimentaria de 54,2% ^39^, al igual que un estudio en Medellín realizado en hogares donde habitaban personas mayores, el cual encontró una proporción de inseguridad alimentaria de 55% ^40^; esta situación agravada por la pandemia del COVID-19, refleja la pobreza o malas condiciones económicas de muchos hogares en los que viven las personas mayores, problemática que debe seguir siendo prioritaria para los gobiernos en los programas de ayudas sociales y alimentarias para los hogares más vulnerables.

La frecuencia de déficit de peso fue más alta a la reportada en el estudio *MaNuEL*, que tuvo en cuenta datos de 11 países europeos, en personas mayores de 65 años que vivían en la comunidad, con un promedio de edad más alto que el nuestro (superior a los 75 años) ^41^. La menor frecuencia de déficit puede deberse al punto de corte que se utilizó para el IMC (< 20kg/m^2^) y que el indicador de pérdida de peso (mayor a tres kilos en el último mes o reducción moderada a severa de la ingesta en los últimos tres meses), puede llegar a tener una subestimación en la respuesta por parte de la población de personas mayores. De igual manera, un estudio en China ^42^, presentó menor porcentaje de déficit (7,18 %), de acuerdo a la definición de desnutrición de la Sociedad Europea de Nutrición Clínica y Metabolismo (ESPEN), esto es, pérdida de peso mayor al 10% en un tiempo indefinido e IMC < 20kg/m^2^ en menores de 70 años o IMC < 22kg/m^2^ en personas de 70 años o más.

Al analizar los indicadores antropométricos para déficit de peso de manera separada, es mayor cuando se utiliza la CB (aproximadamente 53%); mientras que al agrupar la CB con los indicadores IMC y CP, el déficit de peso es menor (aproximadamente 19%), lo que podría sugerir que es más conveniente utilizar las tres medidas agrupadas para lograr un diagnóstico nutricional más preciso, debido a que cada circunferencia aporta información diferente de la composición corporal ^43^.

Los hallazgos de esta investigación demuestran que la condición con síntomas depresivos sobresale en todas las clases latentes, seguida de los problemas de salud oral con presencia en tres de ellas. Estas dos condiciones con relación al estado nutricional de las personas mayores se han investigado por otros autores; en un estudio en Medellín ^44^, se encontró que la malnutrición por déficit de acuerdo al *Mini Nutritional Assessment* (MNA), se asoció con síntomas depresivos (*odds ratio -*OR = 8,08) y con baja percepción de la salud bucal mediante la escala GOHAI (OR = 3,09); otro estudio llevado a cabo en Grecia ^45^, también encontró asociación entre los síntomas depresivos por la escala de depresión geriátrica y la malnutrición por déficit según el MNA (p < 0,001). De acuerdo a varias revisiones, entre ellas la de Αntoniadou & Varzakas ^46^, existe una relación recíproca entre la salud oral y el estado nutricional; refiriendo que la pérdida de piezas dentales tiene implicaciones para la selección e ingesta de alimentos, lo que puede llevar a un bajo consumo de carne, frutas y verduras y, por tanto, menores aportes de fibra y nutrientes específicos como proteínas, calcio y vitaminas A, E, C y ácido fólico; el edentulismo induce a un aumento en la ingesta de alimentos de fácil masticación y de mayor aporte calórico, lo que puede causar un exceso de peso.

Finalmente, una fortaleza de este estudio fue el agrupar dos o tres indicadores antropométricos para la determinación más precisa de la malnutrición en las personas mayores. En cuanto a las limitantes de este estudio: (1) Los datos se tomaron del estudio SABE del 2015 y dadas las condiciones actuales del país, el incremento de la pobreza, algunos cambios sociales y políticos, es probable que la situación nutricional de las personas mayores haya cambiado; sin embargo, los hallazgos siguen reflejando el estado nutricional de las personas mayores de Colombia, como se evidencia en el estudio *Perfil Alimentario y Nutricional* del Departamento de Antioquia 2019 ^6^, localidad que tiene una de las mayores proporciones de este grupo poblacional del país, y que mostró cifras similares de malnutrición a las de SABE, según diversos indicadores antropométricos. (2) No disponer de otros indicadores para valorar el estado nutricional como los bioquímicos y los dietarios de las personas mayores. (3) La recolección de la información fue en un momento del tiempo, por lo tanto, el diseño del estudio no permite hablar de causa-efecto. (4) La precisión en la estimación de algunas categorías de las variables estrato socioeconómico y forma de alimentarse es reducida, dado el bajo número de observaciones, por lo tanto, se recomienda tener precaución en las interpretaciones derivadas de estas dos características.

## Conclusión

La prevalencia de la malnutrición por indicadores antropométricos agrupados en la población adulta mayor de Colombia es considerable. El exceso de peso estuvo determinado por ser mujer, menor edad, valoración alta de la salud oral y ser de estratos socioeconómicos más altos; mientras que el déficit de peso fue mayor en los hombres, de menor edad, con valoración baja de la salud oral y pertenecientes al estrato socioeconómico más bajo. Se recomienda para la evaluación antropométrica la combinación del IMC con las circunferencias de la cintura, del brazo y de la pantorrilla para mejorar la precisión. Además, se sugiere un enfoque diferenciador para el manejo de las personas mayores con malnutrición, porque los factores que condicionan el exceso de peso son diferentes a los que determinan el déficit.

Este es un estudio relevante para la salud pública y la gerontología, porque muestra la prevalencia del exceso y el déficit de peso en personas mayores, combinando diversos indicadores antropométricos y el perfil epidemiológico en estas condiciones.
